# Supplementary figures and images for: Computation of the electroencephalogram (EEG) from network models of point neurons
Source: PLoS Comput Biol. 2021 Apr 2;17(4):e1008893. doi: 10.1371/journal.pcbi.1008893 (PMC8046357; doi:10.1371/journal.pcbi.1008893)

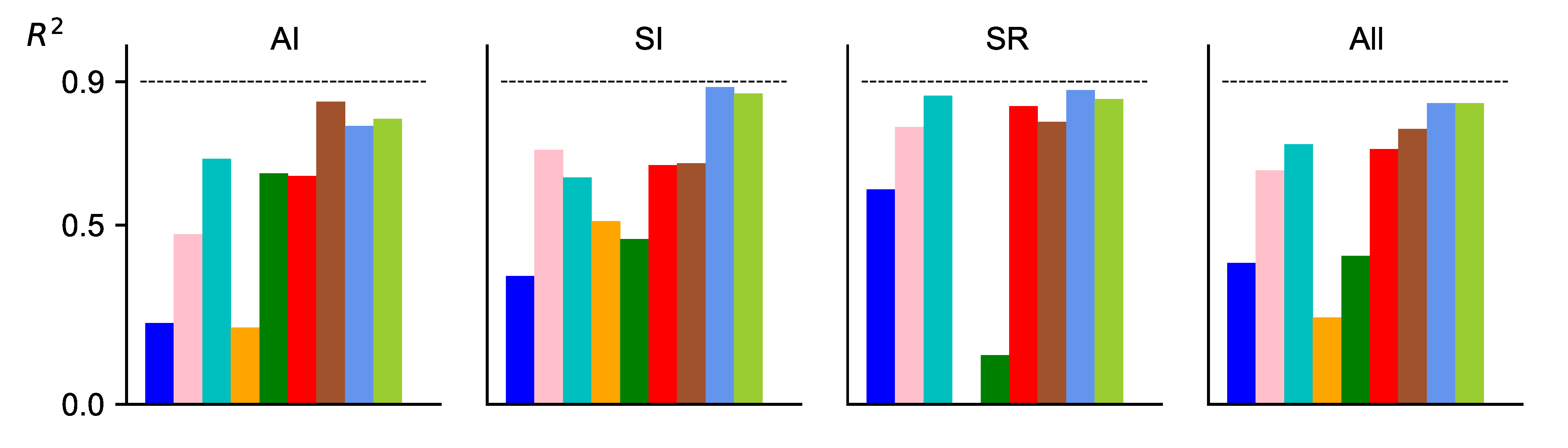

Supplement: S1 Fig — In the same simulation, the “NMC L2/3 PY, clone 9” morphology was randomly assigned to half of the pyramidal-cell population and the “NMC L2/3 PY, clone 0” morphology to the other half. Colors used for proxies are the same used in Fig 4. (TIF) [file pcbi.1008893.s001.tif]

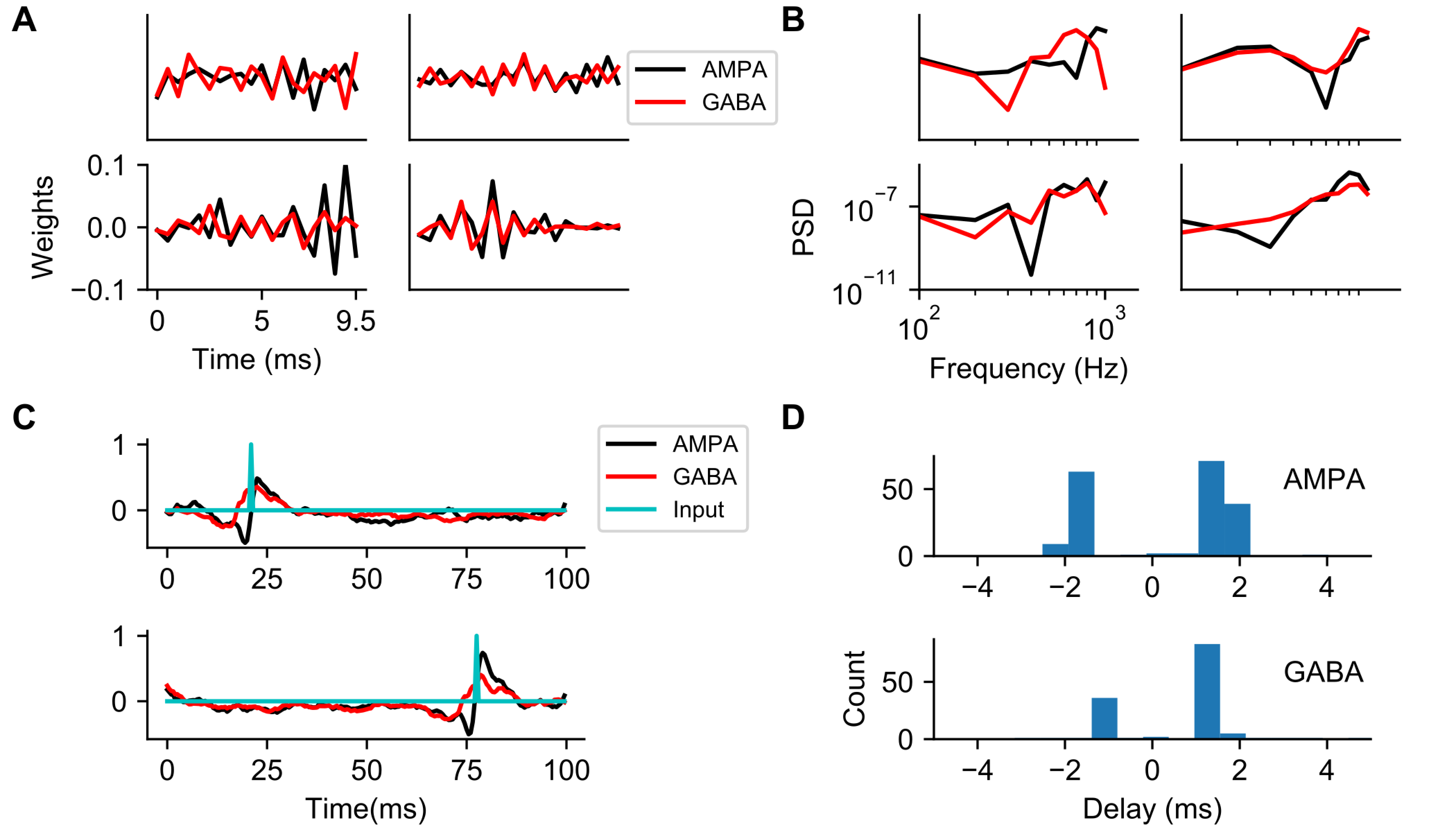

Supplement: S2 Fig — Examples of weights learned by four filters of the convolutional layer, depicted both in the time (A) and frequency domains (B) for the AMPA and GABA inputs. (C) Examples of the CNN outputs in response to unit impulses applied either to the AMPA or GABA inputs. (D) Histograms of time shifts applied to the AMPA and GABA inputs for all combinations of impulses. Each time shift is computed as the difference between the time when the impulse is applied and the time in which the absolute response of the CNN reaches its maximum. (TIF) [file pcbi.1008893.s002.tif]

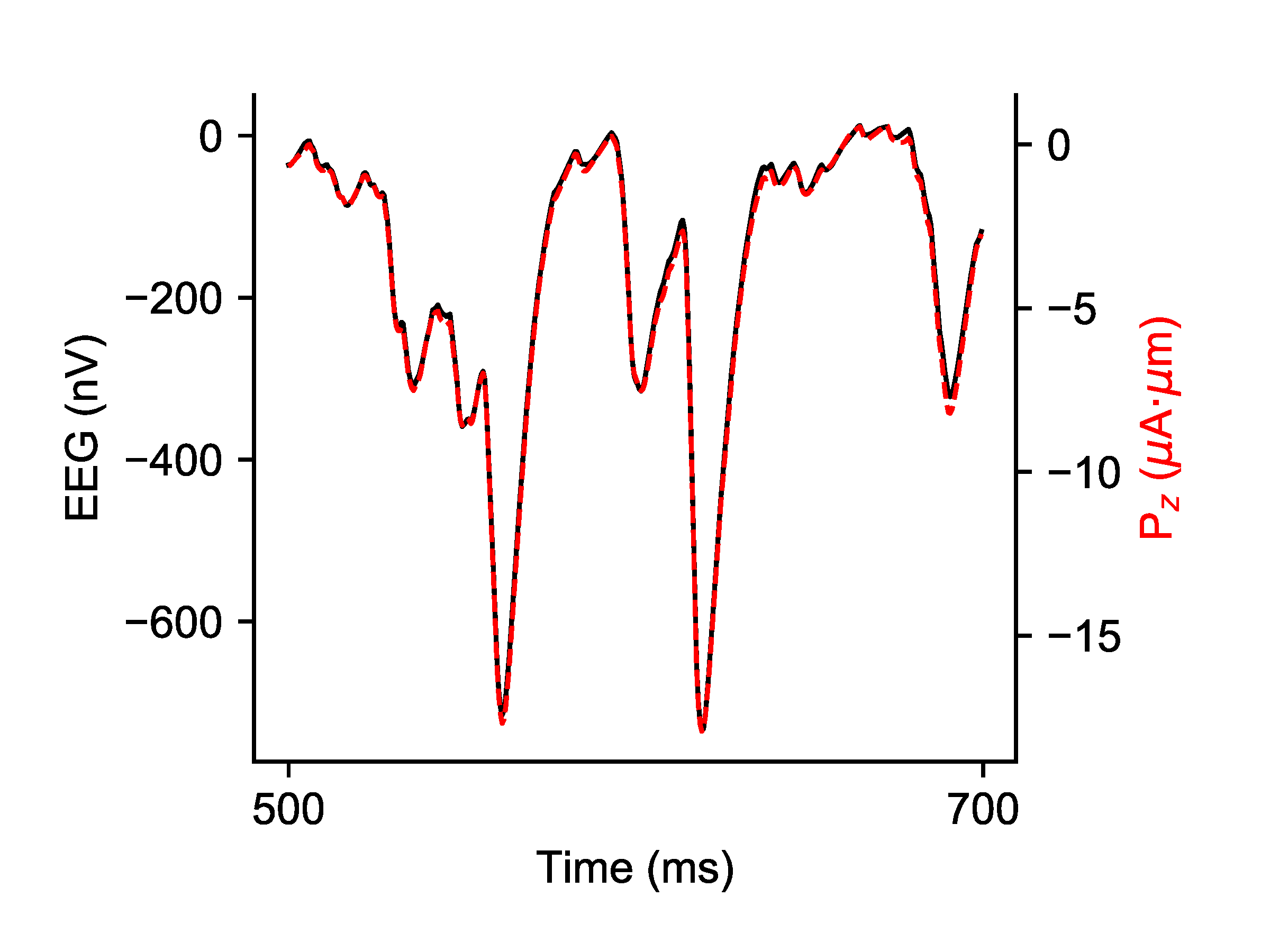

Supplement: S3 Fig — Example of time sequences of EEG (black line) and the dominant component of the current dipole moment, Pz (red dashed line), at the top of the four-sphere head model, computed both on the multicompartment model network. (TIF) [file pcbi.1008893.s003.tif]

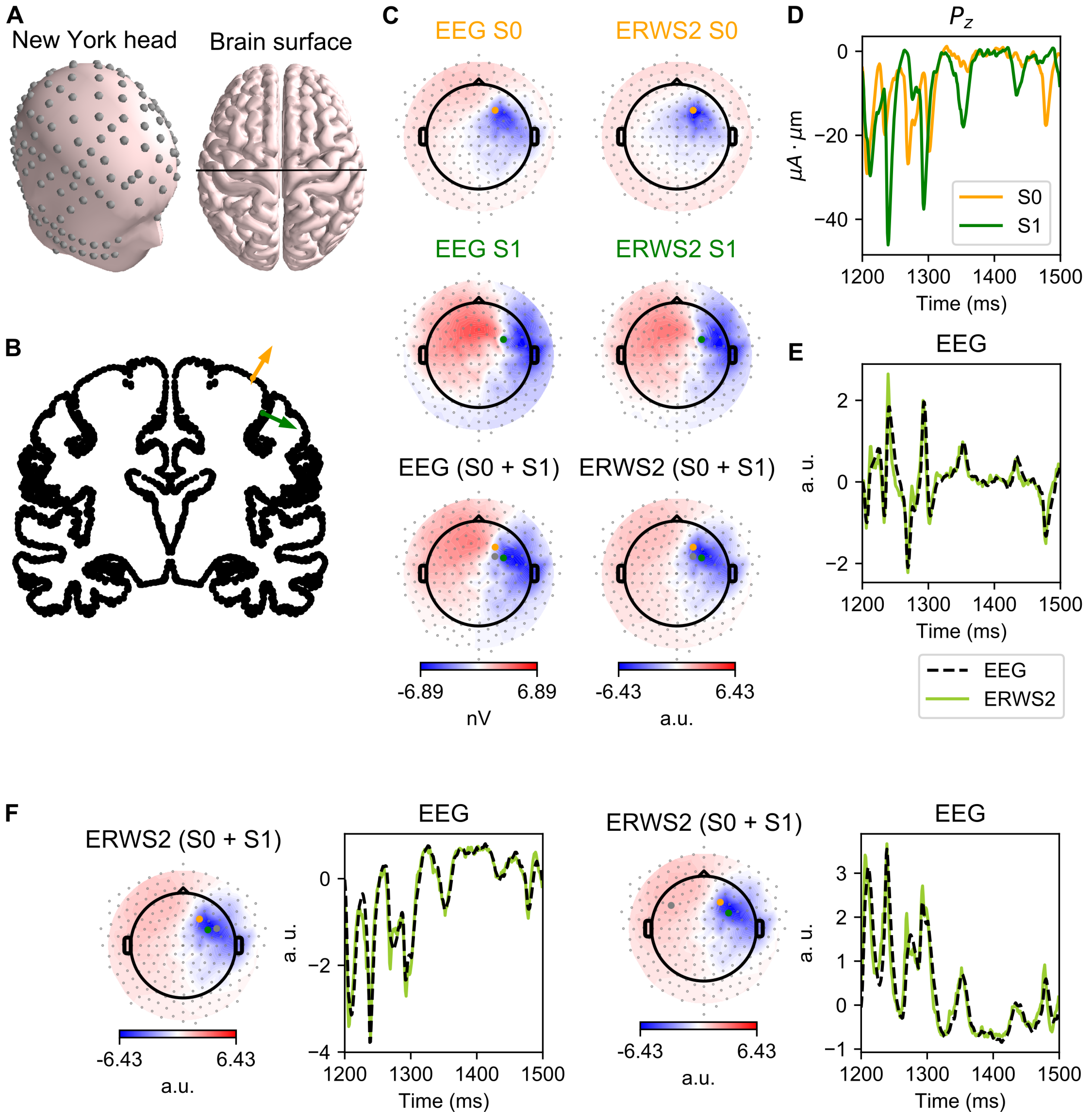

Supplement: S4 Fig — (A) Distribution of EEG electrodes and brain surface of the New York head model [66]. The black line represents the cortical cross-section where current dipoles were placed. (B) Current dipoles of the two subnetworks, S0 and S1, (represented by arrows of different colors) positioned in the cortical cross-section. (C) Topographic maps generated from EEG electrodes projected onto two-dimensional simplified plots of the head model. The ground-truth EEG maps are plotted on the left column and EEG estimations of the ERWS2 proxy on the right column. The closest EEG electrode of each current dipole is plotted as a spot in the same color of the corresponding current dipole. The EEG electrode selected to show time traces of the EEG signal is depicted as a gray spot. (D) Dominant component of the current dipole moment (Pz) for the two subnetworks. (E) Time sequences of the ground-truth EEG and the ERWS2 proxy registered at the electrode shown in panel C. (F) Time traces of ground-truth EEG and the ERWS2 proxy registered at different electrodes located in positions indicated by the gray spots. (TIF) [file pcbi.1008893.s004.tif]
