# Supplementary material for: Computation of the electroencephalogram (EEG) from network models of point neurons
Source: PLoS Comput Biol. 2021 Apr 2;17(4):e1008893. doi: 10.1371/journal.pcbi.1008893 (PMC8046357; doi:10.1371/journal.pcbi.1008893)
Supplement: Attachment — Submitted filename: response2reviewers_PlosComputBiol.docx [file pcbi.1008893.s005.docx]

We have responded to each of the Reviewer’s comments below, and where possible noted down where the changes in the manuscript were done to respond to those comments.

- Reviewers’ comments in **bold font**.

- Author’s reply underneath in normal font.

**Reviewer #1: In this manuscript, Martinez-Cañada and colleagues employ the “hybrid” framework to simulate EEG more efficiently by decoupling the spiking dynamics generation (using point neuron Brunel E-I networks in different regimes) and the generation of electric fields using morphologically realistic, multi-compartmental neurons. The goal of the work is to find suitable proxies measurable in the spiking neural network (SNN), such as population firing rates or synaptic currents, that can be combined to approximate the “ground-truth” EEG signal generated from the biophysical model. The authors compare an array of such proxies and find that optimized lagged summation of the E and I currents performs the best (for linear proxies) across 1) all network regimes, 2) neuron morphologies and dendritic distribution of synapses, 3) EEG electrode positions, and 4) for non-stationary network states triggered by a Gaussian pulse thalamic input. They also test the performance of nonlinear proxies by training a shallow convolutional neural network on the SNN synaptic currents, and find small but consistent improvements over the linear proxies.

Overall, I find this paper and this line of work to be important, since the ability to make physiological inferences from non-invasive human brain signals, such as the EEG, would open up many avenues of research connecting cognitive neuroscience with computational and systems neuroscience. The simulations and analyses performed in this manuscript seem to be of high quality, very comprehensive, and well-documented, and they generate additional insights on the relationships between the different network measurements, such as the delay between firing rate vs. synaptic currents, as well as their relationship with the EEG. That being said, I’m not sure if this manuscript communicates significant advances in insights and tools over what’s already been presented in Mazzoni et al 2015 and Hagen et al 2016 (both of which I am a huge fan of) from the perspective of EEG modeling, due to the validity of the “ground truth” EEG model and a lack of emphasis (in the writing) on the insights that ARE generated from these computational experiments. The latter can be amended in a straightforward manner, for which I have some suggestions writing-wise. The former is discussed as a major concern below, for which I don’t really have recommendations for as a reviewer (other than to conduct non-trivial additional experiments), but I leave it to the editor to evaluate the suitability of this manuscript for publication considering those gaps.**

We are grateful to the Reviewer for these suggestions and insights. As detailed below, we have performed several additional simulated experiments to further test the generality of our results. We also did our best to improve the text by highlighting more the insights on the relationships between EEG and neural activity that come out of our work, and by explaining better the caveats of our work. **The key idea of this paper is that one may use spiking neural network outputs to directly simulate the EEG without computationally expensive biophysical simulations, and these experiments check if and when proxies are good substitutes for the full simulation. As such, my main concern is that the value of this work hinges on the validity of the “ground-truth” EEG model: the more accurate the ground truth EEG model is to real EEG signal generation mechanisms, the more valuable it would be to use an SNN proxy directly. On the other hand, if the ground truth EEG model does not accurately describe known biophysical mechanisms, then it doesn’t matter if one can find perfect proxies using SNN measures to approximate the “ground-truth” simulation, since it would not be useful in realistic situations, especially in use cases suggested in the discussion, i.e., inference. At the very least, this point should be clearly acknowledged in the discussion section as a limitation, beyond what it currently states in terms of extending to more complex head models.**

The Reviewer is suggesting us to improve the study of the validity of the “ground truth” EEG model. Making a 100% detailed and 100% realistic model of an EEG of an individual rodent or human brain is of course out of reach right now and will probably be for decades. What we have been able to do in the revision is something less ambitious but still (in our view) extremely useful: to take the EEG proxy that we developed (optimized on the “training” dataset already described in the previous submission) and test it on EEGs simulated over a wider range of conditions. If EEG proxies are found to work well under this extended set of conditions, it means that even though they are not 100% realistic, they can be reasonably used right “out of the box” under a wide range of conditions. If, in contrast, EEG proxies are found to work badly under this extended set of conditions, we will have learned when to apply and when not to apply it, and this information will be valuable to readers. The specific tests of EEG proxies under a different range of scenarios are reported below, in reply to each specific suggestion of the Reviewer. The main result was that the new EEG proxies worked well when tested on this more extended set of conditions.

In addition to the new simulation results, we have expanded the Section “Limitations and future work” of Discussion, Pages 30-31, to add the caveats suggested by the Reviewer.

**Given that, I feel that both components of the hybrid model suffer weaknesses when compared to real EEG, and I’ve listed the major ones below:

- while the models presented in this work makes some advances from Mazzoni et al 2015, such as including a broader set of Brunel network regimes, I find that the spiking dynamics—and as a result the simulated ground-truth EEG—are not representative of signatures often found in EEG. In the EEG, there is typically strong low-frequency oscillations, such as alpha (10Hz) or beta (20Hz). In addition, the power spectrum is almost always 1/f up to very low-frequencies, and often Lorentzian-like (1/f with plateau at low frequencies). While the simulated EEG here can oscillate at 50Hz in the SR state, which can sometimes be observed in the EEG as gamma oscillations, both the time series and the power spectra do not in general look like realistic EEGs, especially in the SI state PSD (Fig. 1I). I believe this is partially due to the limitations of the Brunel network, as it lacks recurrent dynamics between the cortical and thalamic populations, though that is thought to be a crucial generator of prominent EEG features, such as the alpha rhythms (e.g., Halgren et al., PNAS 2020). Setting up a recurrent thalamocortical network may be outside the scope of the current study, but I suggest at least an experiment using oscillatory thalamic input at 10Hz, similar to what the authors have done with the Gaussian packet to simulate an ERP-like signal.**

Our model of reciprocally connected excitatory and inhibitory populations can produce internally generated gamma oscillations, which are ubiquitously found across brain regions and species and have been associated with several cognitive functions and cognitive disorders. However, we agree that our simple recurrent network model cannot give rise to internally generated oscillations in lower frequency bands, including those commonly observed in EEG/MEG studies - delta (0.5 – 4 Hz), theta (4 – 8 Hz), alpha (8 – 12 Hz) and beta (15 – 30 Hz). As the Reviewer points out, these slower oscillations arise from more complex cortico-cortical and thalamocortical loops, a topic out of the scope of our work. We have added a caveat in Discussion (“Limitations and future work”) in Lines 1002-1013, to highlight that further work will be needed to test our work with broader networks with larger loops that can generate slower oscillations.

In the LIF simulations reported in the previous version of the paper, the LIF network had an “intracortical” input with firing modeled as a broadband Ornstein-Uhlenbeck (OU) process (helping to generate the aperiodic power law component of neural activity) and a “thalamic” input which was constant in time (representing the strength of sensory stimulation of a given sensory cortical patch). Following the suggestion of the Reviewer, we investigated how well the EEG proxies, that we developed and trained using the “Proxy training” dataset of simulations reported already in the previous version of the paper, performed when the LIF network received thalamic inputs with band-limited power added in the canonical low-frequency bands (delta, theta, alpha, beta). This additional power in the thalamic input generated, by entrainment, extra power in the considered band in the EEG generated by the LIF network model (we demonstrated this in earlier publications, see Mazzoni et al. (2008), Plos Comput. Biol., but we replicated this finding in the present paper, (new Fig 8A).

We tried to produce an added oscillatory power that was similar to power values reported in the empirical EEG and MEG literature (Donoghue et al. (2020), Nature Neurosci., He et al. (2018), bioRxiv) in terms of the ratio between added oscillatory power and the aperiodic component of the power spectrum. These empirical studies compute the aperiodic-adjusted power (or flattened spectrum) - that is, the magnitude of the oscillatory peak over and above the aperiodic component. Values of aperiodic-adjusted power of low-frequency oscillations found in older adults for resting-state MEG data (He et al. (2018), bioRxiv) vary approximately in the range (0.05 – 0.3) and in the range (0.1 – 0.5) for EEG data (Donoghue et al. (2020), Nature Neurosci.).

The baseline aperiodic spectrum can be estimated in different ways. One way is to take the EEG power spectrum generated by the same network without added oscillations as a measure of the baseline. The second way is to use an algorithm that can extract the aperiodic component of neural power spectra and apply it to the network with added oscillations. We opted for the latter approach so that we have used the FOOOF algorithm (Donoghue et al. (2020), Nature Neurosci.) to separate the aperiodic component and periodic oscillatory peaks of EEG power spectra allowing us to quantify the relative increase of the oscillatory power in each frequency band with respect to the aperiodic power-law signal.

We then computed the aperiodic-adjusted power. We obtained aperiodic-adjusted power values across the different frequency bands between 0.05 and 0.2 that were within empirical ranges recorded in older adults for resting-state EEG/MEG studies (Donoghue et al. (2020), Nature Neurosci., He et al. (2018), bioRxiv).

When testing our EEG proxies on these new simulations with added power to the thalamic input (not used for training the proxy), we found that our new EEG proxies (both the linear ones and the convolutional ones) still predicted very well the new EEG signal with oscillatory power superimposed on it.

We inserted in the revised paper a new Results Section (“The new EEG proxies are robust to the addition of oscillatory power in canonical low-frequency bands”), Pages 20-22, and a new Figure (Figure 8) that report these new findings. **- the current study only considers AMPA and GABA current, while slower currents, such as NMDA and calcium transients, most likely have strong contributions to the scalp EEG as well, especially since it is thought that low-frequency components of cortical activity sum constructively to make up the EEG. Again, this requires modification to the Brunel network in non-trivial ways, but it is not a guarantee that the current results will hold, or simply extrapolate linearly, with more complex currents, and should at least be mentioned in the discussion.**

We agree that other types of currents may contribute to scalp EEG, such as NMDA currents. However, compared to the large number of LIF modeling studies that have employed AMPA and GABA currents in spiking network models, NMDA currents have not been so well studied. A full understanding of the NMDA dynamics is not available and is beyond the scope of our paper. Since our paper aims to provide modelers who use the well-known simple network model of recurrently connected populations with better mathematical methods to compute EEGs than previous methods, we think that spending too much of our time on this extension would be of limited impact.

To address the concern of the Reviewer, we have acknowledged this limitation and over-simplification of our work in the Section “Limitations and future work” of Discussion, in Lines 1025-1028. In this paragraph, we also include the development and testing of proxies that include NMDA contributions as an area that will need further work.  **- neither the SNN nor the biophysical column have distance-dependent connections. I’m not sure how big of a deal this is, especially since the point neuron identities are randomly assigned to the biophysical neurons, but spatial correlation of activity could be another factor that would change how the sources are summed to get the EEG.**

A full study of how the performance of the proxy depends on the spatial structure of the connections is out of scope and would be unfeasible in this revision. However, in our spatially extended version of the network model (see response to the next point for more details), we have performed proof-of-concept experiments. We divided a spatially more extended network into spatially separated subnetworks and we have studied the performance of proxies in two limiting cases of connectivity patterns between subnetworks (all-to-all or unconnected) that are at different ends of the spectrum of possible spatial connectivity patterns. We have shown in these new simulations that the EEG proxy combining the activity of the different subpopulations still leads to an excellent EEG prediction (new Fig 9).

The new results are reported in a new figure (Fig 9) and are described in a new Results Section (“Upscaling of the network model and integration of EEG by proxies calculated on local subnetworks of cells separated across space”) on Pages 22-25.

In the revision, we also acknowledged that more exhaustive studies that include realistic distance-dependent connectivity are necessary to provide accurate measures of the relative contribution of local proxies to EEGs as a function of cortical distance. This limitation of our work has been added to the Discussion (“Limitations and future work”) in Lines 1029-1037. **- the ground-truth EEG signal is assumed to come from just a single cortical column, and while this is closer to the truth for the extracellular LFP, it’s not accurate as a model of the EEG. It’s more likely that the signal an EEG sensor picks up is a complex summation of many such cortical columns across centimeters, oriented in different directions due to cortical folding, and even from heterogeneous cell-types, ratios of E/I cells, and neuronal morphologies (see. e.g., Wang Nat Rev Neurosci 2020). While a full-cortex spiking neural network model with heterogeneous populations is obviously out of the scope, it would be nice to see multiple interacting cortical populations spaced farther apart to mimic source mixing in EEG, especially considering the hybrid model studied in Hagen et al CerCor 2016. Or, at the very least, acknowledge the need to consider such factors in the discussion.**

We thank the Reviewer for this useful suggestion. We agree that the EEG is a complex signal that integrates neural activity from different cortical columns with different orientations, with heterogeneous cell populations and neuronal morphologies and even with different ratios of E/I cells. Understanding the role played by all these factors that give rise to the features of the EEG is very much an open area of research and more experiments, analyses, and models are necessary to answer this question.

Our work can be seen as a first step to effectively modelling the contribution to the EEG of a two-population network model within one cortical column (which might be considered as the “basic building block” of a multiscale neural network model). Since, by the laws of electromagnetism, electrical potentials in the brain tissue add linearly, we expect the total EEG to be approximated by a suitable linear combination of individual proxies computed for each population, in each cortical column with a specific orientation. In the results reported already in the previous version of the paper (Fig 4) and in new simulations added now (S1 Fig) we have also demonstrated that our proxies are robust to different cell morphologies and work well also with populations with heterogeneous morphologies. Therefore, we hypothesize that, in more complex models with multiple cortical columns and heterogeneous cells populations, the total EEG could be in principle straightforward to compute using our proxies if the amplitude of each laminar contribution is known.

In the revised manuscript, we ran some initial proof-of-concept simulations to test these hypotheses. Specifically, we developed an upscaled version of the network model that contains four times more neurons, i.e., 20000 neurons, preserving the density of neurons and local synapses to generate similar network dynamics. With this spatially extended model, we performed some initial investigations on how proxies could integrate the EEG signal across points in space.

We divided the whole population of cells into 4 spatially distinct subnetworks. We considered two simplified scenarios of connectivity that are at opposite ends of the spectra of possible connectivity between subnetworks: either the 4 subnetworks are all-to-all connected or they are unconnected. Local proxies computed for each subnetwork of cells were compared with the global proxy, which was calculated as the linear sum of all local proxies. In the model with all-to-all connectivity, computation of proxies from local subnetworks of cells separated across space could be used to produce accurate estimations of the EEG (new Fig 9). In the unconnected network, we found that local proxies could only partially approximate the EEG and the prediction performance significantly dropped, in particular for faster network dynamics (i.e., SI and SR states). However, the linear sum of proxies computed on all subnetworks provided an excellent approximation to the EEG, similar to that obtained with the all-to-all connectivity (new Fig 9).

To study the influence of different orientations of cortical columns on the EEG, we evaluated, as an example, the performance of our proxies in approximating the EEG produced by two orthogonally oriented neuronal populations. For this purpose, we computed EEGs on a more complex head model that accounts for the folded cortical surface of the human brain (The New York head model, see response to the next request for more details). We placed two current dipole moments generated by different subnetwork populations in two different locations spatially separated and orthogonally oriented on the folded cortical surface of the New York head model. We computed EEG signal at all locations of the EEG electrodes for the two dipoles. The total EEG map was calculated as the sum of individual EEG maps generated by each current dipole. We show in the new version of the manuscript that the EEG can be fairly well approximated by our proxies even in this scenario with mixing orientations of the EEG sources.

We added new Sections (“Upscaling of the network model and integration of EEG by proxies calculated on local subnetworks of cells separated across space” and “Evaluation of proxies in a human head model”) on Pages 22-27 that report these proof-of-concept results.

**- similarly, the head model is a simple 4-layer spherical model with differential resistance, which is arguably the only addition to the model from Mazzoni et al PLOSCB 2015. The authors state in the discussion that more complex head models can be easily incorporated, since the simulated EEG sensors essentially pick up a scaled version of the dipole in the z-direction. However, as one of the main result is that the proxy perform differently when the sensor is at a different angle with respect to the population, and that cortical folding would imply the summation of sources from differentially oriented pyramidal populations, I’m not convinced that ERWS, for example, would apply straight out the box in that situation. It would be more convincing to show this with several columns, or discussed as a limitation.**

As already introduced in the reply to the above request, we computed EEGs on a highly detailed model of the individual human anatomy that incorporates 231 electrode locations: the New York head model (Huang et al. (2016), Neurorimage). We computed the current dipole moments of two different subnetworks from the unconnected network model and we placed them in two different locations spatially separated and orthogonally oriented on the folded cortical surface of the head model. We computed EEG signals at all locations of the EEG electrodes for the two dipoles. The total EEG map was calculated as the sum of individual EEG maps generated by each current dipole. We report in the new version of the manuscript that the EEG can be well approximated by our proxies even in this scenario with mixing orientations of the EEG sources (new S4 Fig).

We wrote a new Section of Results that report these findings: “Evaluation of proxies in a human head model”, on Pages 26-27.

**- relating to the previous point, I think it’s not clear until much later on (first time in L191) that the EEG model is meant to be a rodent model (with a downscaled and smooth cortex). I think it would orient the reader to have the correct expectation if this was mentioned as early as possible, potentially even in the abstract.**

We revised abstract and Introduction, as well as specific parts of Results, to make it clear that the bulk of our modelling work is based on rodent data. **some stand-alone comments:

- I believe that the manuscript would be improved if specific findings are more explicitly highlighted, perhaps even in the abstract. For example, the fact that linear combination of AMPA/GABA currents is better than firing rate or Vm (e.g., L674) is very important for somebody simulating EEG signals from spiking networks, since firing rate is very commonly (and perhaps mistakenly) used as a surrogate. Also, it’s quite interesting that the optimized parameters for ERWS proxies are state-dependent, even though the physical shapes of the neurons themselves are unchanged, which suggests frequency-dependent filtering characteristics of the membrane or synapse.**

With regard to the fact that a linear combination of AMPA/GABA currents is a better proxy than using firing rates or Vm, we highlighted it in the abstract. We created a new subsection of Discussion “Simulations of EEGs using average membrane potentials or average firing rates are less accurate than using synaptic currents” that better highlights the significance of this important result (Page 27).

With regard to the second issue, we believe that the state-dependent relationship of parameters of ERWS2 is due to the properties of the synaptic currents. Our model uses realistic synaptic currents and it is based on conductances and not on currents (as often done in simpler and less realistic work, see e.g. the original classic papers of Brunel and Wang).

In the conductance-based model, the synaptic current is:

$I_{syn}\left( t \right)=g_{syn}s_{syn}(t)\left( V_{m}\left( t \right)-E_{syn} \right)$

Where $g_{syn}$ and $E_{syn}$ are respectively the conductance and the reversal potential of the synapse. $s_{syn}\left( t \right)$ describes the time course of the synaptic current. Unlike in the current-based model, AMPA and GABA currents of the conductance-based model depend on the membrane potential $V_{m}$ through the driving force $\left( V_{m}\left( t \right)-E_{syn} \right)$, which describes the varying strength of the synapse. It was shown in previous simulations of networks of point-neurons that the mean membrane potential decreases when the external rate increases (Cavallari et al. (2014), Frontiers in Neural Circuits). Consequently, the driving force of GABA currents will be reduced because $V_{m}$ gets closer to the reversal potential of GABA, while the driving force of AMPA currents will increase. In agreement with this reasoning, in the ERWS2 proxy, we observed a larger weight of GABA currents for low values of the external input and the opposite effect, stronger weight of AMPA currents, as the external rate increases. Of course, this is true both for the AMPA and GABA currents calculated from the point-neuron network (and used as input to the proxy), and for the AMPA and GABA currents that arise from projecting the spikes onto the multicompartment neurons. However, the membrane potential may be much more variable in the point-neuron simulations (since all synapses are at a single point), than in the multi-compartment simulations. If so, this could introduce a dependence of the state in the proxy.

We created a new subsection of Discussion “State-dependent relationships between predicted EEG and neural activity”, in Page 28, that includes this reasoning.

**- computing the R^2 on the time series emphasizes low-frequency fluctuations due to 1/f scaling of EEG signals, does this potentially obfuscate important differences in performance for the different metrics?**

Following another suggestion below of this Reviewer, we revised the calculation of R^2 in the frequency domain (Fig 2J) to weight power spectra on log power. When doing so, we found results for proxy performance that match those of the time domain much better than before and that show differences across proxies in the spectral domain performance (the previously reported analysis over-emphasized the errors at lower frequencies, and therefore did not show interesting patterns of performance across proxies). We added some text in Results (Lines 405-411) to compare results in the frequency and in the time domain. We also separated the analyses for different network states, which have different frequency components, and in the revision we also manipulated the frequencies in the external input. Thus, we feel that the effect of the frequency composition of network activity on the performance of the EEG proxy is much better addressed in the revision, and we are grateful to the Reviewer for suggesting this.  **- R^2 = 0.9 is used as a benchmark throughout the study, but why, and what is considered “good”?**

The R^2 = 0.9 value as reference for good performance is arbitrary and is used only to aid a rapid visual inspection of the results. We added a line to the caption of Figure 2F (the first panel in which this line is used) to make this explicit.  **- is the PSD R^2 computed on log power or raw power? To complement the time series R^2 comparison, perhaps it would be good to compute R^2 on the logged power to de-emphasize the low frequencies.**

The PSD R^2^ was computed on the raw power spectrum. We now calculated R^2^ on log power and new results have replaced previous panel J in Fig 2. We thank the Reviewer for pointing out the need to use the logarithmic scale of the power spectrum to emphasize differences in performance between the different proxies. We added the following information to the text of the revised manuscript. (Page 12, Lines 405-407):

“We logarithmically weighted the spectra to prevent R^2^ to be dominated by low frequencies, neglecting the importance of errors at high frequencies.” **- worth discussing why Fig1G (R^2 on time series) and J (R^2 on PSD) are so different**

After computing R^2 on the log power spectrum, we now obtained much more similar results of R^2 on both time and spectral domains. We revised the results text, Lines 405-411, to describe the new results.  **- L361: why are FR, sum(I) and Vm so bad? Apologies if I missed the extended discussion on this but I think this is an interesting point, further supporting the insight that the EEG is more about absolute current fluctuations, and less so about net input.**

Part of the reason why FR and Vm are relatively bad proxies, has to do with time-shifts, and in Fig 3 we already demonstrated that finding an optimal time shift can substantially increase the accuracy of these two proxies. Additionally, the EEG is caused by transmembrane currents from synaptic inputs, and it is well known from theory that both membrane potentials and firing rates simply have different temporal dynamics than the transmembrane currents. We can therefore expect best proxy to depend on the synaptic input currents.

This was addressed in our manuscript (Lines 430-436), where we wrote:

"Two proxies, FR and Vm, clearly improved the quality of their fits after time shifting but presented opposed time shifts: while FR was delayed, Vm was moved forward in time. A spike is a local and instantaneous event in time and, as a result, a firing-rate proxy is expected to exhibit faster temporal changes than the EEG signal. By contrast, somatic integration of the postsynaptic membrane potentials following presynaptic spiking is a slower process that might lead to a signal more low-pass filtered than the EEG."

There is, however, probably more to it: FR only represent the firing rate of the excitatory neurons. Inhibitory neurons can however also be expected to have a large effect on the EEG signal, but only through their synaptic inputs onto pyramidal cells (Hagen et al. (2016), Cerebral cortex; Teleńczuk et al. (2016), Scientific Reports). Importantly, these inhibitory synaptic inputs will be reflected in the synaptic currents (and therefore in ERWS1/2), but not in FR (other than through an indirect effect on the pyramidal cells firing rates). For FR to be a good proxy, it would probably also need to include the firing rates of the inhibitory neurons, but since the firing rate of the inhibitory neurons and excitatory neurons can be expected to have completely different effects on the EEG, the FR proxy would therefore probably need to be an optimized weighted sum of excitatory and inhibitory firing rates. However, even in this case, as noted in our manuscript (pasted in above), we would expect FR to have a somewhat too fast temporal dynamics.

With regards to Sum(I): as illustrated in Figure 1C, AMPA and GABA currents are differently distributed on the neural morphologies, based on biological data. This means that they, on average, will generate different patterns of transmembrane currents, which again means that we can expect AMPA and GABA currents to differently affect the EEG signal. Therefore, an optimized weighted sum of the currents is a much more sensible approach than a simple sum of these currents.

We revised the Discussion to add a short subsection specifically devoted to this issue (“Simulations of EEGs using average membrane potentials or average firing rates are less accurate than using synaptic currents”) on Page 27.  **- in my opinion, Fig 8 complicates the main message, which I interpret to be that linear proxies are sufficient for estimating the EEG, and thus can be moved to be supplemental as additional analyses.**

We moved this figure to the Supporting information (new S2 Fig), as suggested.

**To summarize, while the EEG ground-truth model realism is a matter of the authors’ decisions, and one can always include more details at the cost of computational complexity, there are several key factors that should be considered, or at least addressed and justified as to why they are omitted. On the other hand, the dynamics produced in the ground truth simulation do not resemble realistic EEG, and while I don’t think a computational study MUST include empirical data, I do think comparison to some real data, even just at a surface level, would ground this work, especially considering the implications suggested in the discussion section regarding inference. I absolutely agree that computational models serve as an important tool for understanding the EEG and allows for physiological inferences. However, while the discussion currently (L730-744) is a nice exposition of the potential uses in theory, since this paper does not actually do it with real data, it’s unclear then from the paper itself how the current framework is suppose to be used for a naive reader (like if an EEG practitioner that wants to use the simulations for inference). So the claims read a little empty. Furthermore, some key references that work on those approaches, such as the Human Neocortical Solver (Neymotin et al eLife 2020), are missing.**

In the section of Discussion “Possible applications and impact of the new EEG proxies”, from Line 929, we explain more explicitly how our EEG proxies could be used to better infer network parameters from real EEG data. We also clarify that this extension is for future work and was not attained here. We also added citations to the work of Neymotin and colleagues and Sanz Leon and colleagues on Line 951.

In addition, to make room for all the new additions indicated above, we trimmed some sentences of the Abstract and Discussion without changing the meaning.

**Reviewer #2: Building upon previous work to introduce a proxy for the LFP generated by a network of point neurons, in this work the authors adapt this approach to develop a new proxy for the EEG generated by a network of point neurons. As in previous work, the authors feed spikes generated in the point neuron network to a multicompartment, morphological model in order to compare LFP generated by the point neuron network to a ground truth. They use this approach to develop a new proxy suitable for the EEG generated from a network of neurons. This new proxy surpasses previous work, in that this proxy remains valid across a wide range of network states. Finally, they extend their approach using a shallow CNN to understand how much more variance could be explained by more complicated nonlinear proxies.

This manuscript reports well-executed and important work. The utility of a well-motivated EEG proxy in the field of neuroscience is great. There are a few revisions and minor suggestions to address in this round of review.**

We are grateful to the Reviewer for their kind comments and useful suggestions, which helped us to improve our work.
 **MAJOR

- line 153: The parameters for the LIF model are well documented in the tables; however, it would be helpful to have a little information in the main text about the approximate scale (i.e. number of neurons and synapses) when introducing the LIF model.**

We have now added the following text on Page 5, Lines 162-167.

“The network was composed of 5000 neurons: 4000 were excitatory (i.e., their projections onto other neurons formed AMPA-like excitatory synapses) and 1000 inhibitory (i.e., their projections formed GABA-like synapses). The neurons were randomly connected with a connection probability between each pair of neurons of 0.2. This meant that, on average, the number of incoming excitatory and inhibitory connections onto each neuron was 800 and 200, respectively”

**This also raises an important point: what network scale is sufficient to faithfully model the EEG? Would the network dynamics change significantly for more than 5,000 neurons, and would this have an impact on the simulated EEG? Lastly, in the case that a larger network would need to be considered, how could the EEG proxy integrate signal across points in space?**

We thank the Reviewer for these useful suggestions. In the revised manuscript, we ran some initial proof-of-concept simulations to address these points.

We developed an upscaled version of the network model that contains four times more neurons, i.e., 20000 neurons, preserving density of neurons and individual synapses to each neuron. With this spatially extended model, we performed some initial investigations on how proxies could integrate the EEG signal across points in space (new Fig 9).

We divided the whole population of cells into 4 different subnetworks. We considered two opposite scenarios of recurrent connectivity: either the 4 subnetworks are all-to-all connected or they are unconnected. Local proxies computed for each subnetwork of cells were compared with the global proxy, which was calculated as the linear sum of all local proxies. In the model with all-to-all connectivity, as expected, network dynamics were similar between subnetworks. We found that computation of proxies from local subnetworks of cells separated across space could be used to produce reliable estimations of the EEG. In the unconnected configuration, network dynamics were different between subnetworks and local proxies could only partially approximate the total EEG. In this case, the prediction performance of local proxies dropped significantly, in particular for faster network dynamics (i.e., SI and SR states). However, the linear sum of proxies computed on all subnetworks provided the same patterns of performance obtained with the all-to-all connectivity.

We added a new Results Section (“Upscaling of the network model and integration of EEG by proxies calculated on local subnetworks of cells separated across space”) on Pages 22-25 that report these results.

**- line 448: It is useful to note that the parameter values for the proxies were fixed across different morphologies. This increases confidence that the EEG proxy will generalize well across different morphologies. One point, however, is that the same morphology is applied for the pyramidal cells and the interneurons in the full simulation. What effect could heterogeneity in neuronal morphologies have on the proxy comparison? Is there some way to show that this will be negligible?**

The Reviewer is right to note that proxies were trained using only one type of pyramidal-cell morphology in each simulation case, although different morphologies were individually evaluated in each simulation (see Fig. 4). In other words, in the previous version of the manuscript we tested the EEG proxy on EEGs generated by a homogenous population of neuronal morphologies. We now extended this work to test the proxy also on EEGs generated by a heterogeneous population of pyramidal-cell morphologies. We found that all main results were confirmed also in this case, with our new EEG proxies working well and working much better than V_m_- or FR-based proxies. We also found that performance scores of proxies were between the values obtained independently for each morphology.

These results are reported in new S1 Fig and described in Results, Page 14, Lines 496-503.

**- Fig 6: The results from this point are important but somewhat difficult to interpret. What do these distances mean in terms of typical EEG placements in a human experiment?**

These distances are related to a rodent head model, which is the model used in our work, and have nothing to do with distances used in a human experiment because of the difference in scale. We made it more explicit in the revised paper (abstract and Introduction) that the head model used for simulations is a rodent head model and we have provided the reason that justifies it.

Additionally, we computed EEGs on a highly detailed model of the individual human anatomy that incorporates 231 electrode locations: the New York head model (Huang et al. (2016), Neuroimage). Placements of EEG electrodes in this head model correspond to realistic distances in a human experiment. We have shown that our proxies can still approximate well EEGs at different electrodes distantly separated.

These new results are described in the new Results Section “Evaluation of proxies in a human head model”, Pages 26-27 and new S4 Fig. **- Lastly, this proxy has clear potential to shed light on important signals recorded in EEG, for example sleep rhythms. Could this proxy already give some insight into oscillations recorded in the sleep EEG?**

Thanks for this suggestion. As discussed in more length to the point of Reviewer 1 about adding slower oscillations, our LIF recurrent network cannot intrinsically generate slow oscillations, which would require much more complex networks with thalamocortical and cortico-cortical loops. As such, our current simulations cannot offer new insights on the generation of sleep rhythms. However, as reported above in reply to the point of Reviewer 1, we now tested the performance of our proxies in new simulations obtained by increasing the power of the thalamic input in some canonical low-frequency bands (delta, theta, alpha, beta) commonly analyzed in EEG/MEG studies. We found (new Fig 8) that our EEG proxies work very well with oscillations in each canonical band, including the delta band which contains slow waves of NREM sleep, suggesting that our proxies could be also successfully applied to models able to generate slow oscillations.

We inserted in the revised paper a new Results Section (“The new EEG proxies are robust to the addition of oscillatory power in canonical low-frequency bands”), on Pages 20-22, and a new Figure (Fig 8) that reports these new findings. **MINOR

- In general, the notation in the equations could use improvement. Several style guides caution against multiletter abbreviations for mathematical variables (cf. Physical Review Style and Notation Guide, Section C, Point 4), as it makes equations less clear.**

We thank the Reviewer for this suggestion. We now corrected all equations to replace mathematical variables that used multiletter abbreviations with conventional symbols instead, such as $I$ for synaptic currents or $\Phi$ for the proxy (which is intended to describe an electric field potential).
 **- line 539: Are these "parameters" or "state variables"?**

It refers to the variables of the LIF network model. “Parameters” has been changed to “variables” (of the LIF network model) on Line 575. **- line 562: What is the unit of the standard deviation?**

We defined the prediction error as the difference between amplitude values of the predicted and true EEG signals at a specific time step of the simulation. Since the predicted and the true EEG signals are z-scored, the prediction error is also z-scored, and their units are Standard Deviation Units (SDU). The standard deviation is expressed in the same units as the observed magnitude, which are Standard Deviation Units (SDU).

We modified the caption and panel C in Fig 7 of the new version of the manuscript to indicate that the prediction error is expressed in SDU. **- Fig 7D: What is the unit of "True values" and "Predictions"?**

According to the previous response, they are SDU. **- line 606: This sentence is unclear. Is there a typo?**

We replaced this sentence with following new one: “The CNN includes layers that were non-linear besides the first convolutional layer”, on Lines 638-639.

**Have all data underlying the figures and results presented in the manuscript been provided?
Large-scale datasets should be made available via a public repository as described in the *PLOS Computational Biology*** [**data availability policy**](http://journals.plos.org/ploscompbiol/s/data-availability)**, and numerical data that underlies graphs or summary statistics should be provided in spreadsheet form as supporting information.**

**Reviewer #1: Yes**

**Reviewer #2: None**

The source code to simulate the LIF and multicompartment model networks can be downloaded at https://github.com/pablomc88/EEG_proxy_from_network_point_neurons. Datasets generated in this study, scripts to plot the figures and weights of the CNN are available from http://doi.org/10.5281/zenodo.4506494.

This information has been included in the Section “Data and Code availability” of the manuscript (Page 41).

**Figure Files:**

**While revising your submission, please upload your figure files to the Preflight Analysis and Conversion Engine (PACE) digital diagnostic tool,** [**https://pacev2.apexcovantage.com**](https://pacev2.apexcovantage.com/)**. PACE helps ensure that figures meet PLOS requirements. To use PACE, you must first register as a user. Then, login and navigate to the UPLOAD tab, where you will find detailed instructions on how to use the tool. If you encounter any issues or have any questions when using PACE, please email us at** [**figures@plos.org**](mailto:figures@plos.org)**.**

We checked the files on PACE as suggested

**Data Requirements:**

**Please note that, as a condition of publication, PLOS' data policy requires that you make available all data used to draw the conclusions outlined in your manuscript. Data must be deposited in an appropriate repository, included within the body of the manuscript, or uploaded as supporting information. This includes all numerical values that were used to generate graphs, histograms etc.. For an example in PLOS Biology see here:** [**http://www.plosbiology.org/article/info%3Adoi%2F10.1371%2Fjournal.pbio.1001908#s5**](http://www.plosbiology.org/article/info%3Adoi%2F10.1371%2Fjournal.pbio.1001908#s5)**.**

The Data and Code Availability section (Page 41) was updated to include the URL of the data and software repositories. The source code to simulate the LIF and multicompartment model networks can be downloaded at https://github.com/pablomc88/EEG_proxy_from_network_point_neurons. Datasets generated in this study, scripts to plot the figures and weights of the CNN are all publicly available from http://doi.org/10.5281/zenodo.4506494.

**Reproducibility:**

**To enhance the reproducibility of your results, PLOS recommends that you deposit laboratory protocols in protocols.io, where a protocol can be assigned its own identifier (DOI) such that it can be cited independently in the future. For instructions, please see** [**http://journals.plos.org/compbiol/s/submission-guidelines#loc-materials-and-methods**](http://journals.plos.org/plospathogens/s/submission-guidelines)

Not relevant for this paper
