# Supplementary material for: Computation of the electroencephalogram (EEG) from network models of point neurons
Source: PLoS Comput Biol. 2021 Apr 2;17(4):e1008893. doi: 10.1371/journal.pcbi.1008893 (PMC8046357; doi:10.1371/journal.pcbi.1008893)
Supplement: Attachment — Submitted filename: response2reviewers_PlosComputBiol.docx [file pcbi.1008893.s006.docx]

We have responded to each of the Reviewer’s comments below, and where possible noted down where the changes in the manuscript were done to respond to those comments.

- Reviewers’ comments in **bold font**.

- Author’s reply underneath in normal font.

**Reviewer #1:**

**dear all,**

**many thanks to the authors for engaging so constructively with my comments. I read with great interest the additional experiments and insights they generated, as well as the extensive explanations the authors provided (e.g., on conductance vs. current based models and their implications near reversal potential, timing differences of currents vs. Vm, etc.), which I learned a lot from. As previously agreed upon, these set of experiments are satisfactory to me, and I believe they extend the study in many directions than originally presented, and I hope the authors feel the same after the great efforts after the first round of reviews. The additional text also provide better framing and more explicit acknowledgement of the limitations (and therefore future opportunities) of this work, and I agree that this is an excellent step towards human EEG signals with biophysical modeling.**

**just one small note: the PSDs in figure 8 look a bit strange, in that none of them show clear oscillatory peaks except in perhaps the delta/theta range, while the aperiodic-adjusted power was apparently non-trivial (as the authors state, compared to results from human data in literature). I'm wondering if there was potentially a mix up with the PSDs plotted? If not, it is curious that the network then somehow filtered out the input frequencies (at least by visual inspection from the PSDs). This does not hinder acceptance in my view, but could be good to discuss, especially as curious readers may wonder where those oscillatory inputs have gone.**

**again, excellent work and it was a pleasure to be a small part of this process**

**best,**

**richard gao**

We would like once again to express our gratitude to the Reviewer for the suggestions and insights. We have revised Figure 8a as discussed. In particular, we removed the full model fit line, which could confuse the readers and mask the position of the PSD peaks. We also emphasized with a vertical blue line the position of the PSD peak for each band.
